# Novel insights into the synergistic interaction of Bortezomib and TRAIL: tBid provides the link

**DOI:** 10.18632/oncotarget.277

**Published:** 2011-05-16

**Authors:** Simone Fulda

**Affiliations:** ^1^ Institute for Experimental Cancer Research in Pediatrics, Goethe-University Frankfurt, Komturstr. 3a, 60528 Frankfurt, Germany

**Keywords:** cancer, apoptosis, chemotherapy, proteasome inhibitor, TRAIL

## Abstract

The proteasome inhibitor Bortezomib has been identified as a potent enhancer of TRAIL-induced apoptosis in several human cancers. However, the identification of the underlying molecular mechanisms of this synergistic cell death induction has been ongoing over the last years. A recent study identifies a new mechanism of action for the synergism of TRAIL and Bortezomib.

Evasion of apoptosis (programmed cell death) belongs to the hallmarks of cancers and contributes to tumor progression and treatment resistance [1]. There are two major apoptosis signaling pathways that culminate in the activation of caspases, i.e. the death receptor (extrinsic) pathway, which is triggered by the ligation of death receptors at the cell surface, and the mitochondrial (intrinsic) pathway, which involves the release of apoptogenic proteins from mitochondria into the cytosol to initiate caspase activation [2]. In principle, agents that trigger agonistic TRAIL receptors such as TRAIL receptor antibodies or recombinant soluble TRAIL present promising cancer therapeutics, since they can directly initiate the apoptotic machinery in cancer cells [3]. However, many human cancers have developed mechanisms to escape the induction of apoptosis upon treatment with TRAIL [4]. This underscores the need to identify and validate novel agents that could be used in combination protocols with TRAIL receptor targeting agents to potentiate the antitumor activity of TRAIL-based regimens.

Inhibition of the proteasome presents one such strategy to enhance the sensitivity of cancer cells towards TRAIL. For example, Bortezomib (PS-341, VELCADE) is a dipeptidyl boronic acid compound that reversibly blocks the proteolytic activity of the proteasome and is a FDA-approved drugs for the treatment of multiple myeloma [5]. While Bortezomib has been shown to increase the sensitivity to TRAIL-induced apoptosis in several human cancers as single agent and in combination protocols [6, 7], the identification of the underlying molecular mechanisms that are responsible for this synergistic induction of apoptosis has been the subject of intensive investigations over the last years.

A recent study identifies a novel mechanism of action that underlies the synergistic cooperation of TRAIL and Bortezomib by demonstrating for the first time that Bortezomib enhances the stability of TRAIL-derived tBid, the cleaved form of Bid [8]. Bid is a pro-apoptotic BH3-only domain protein of the Bcl-2 family [9]. Upon stimulation of death receptors such as TRAIL receptors or CD95, activation of caspase-8 at the death-inducing signaling complex (DISC) results in the proteolytic processing of Bid into tBid [2]. tBid in turn translocates to mitochondria to promote activation of Bax and Bak, cytochrome c release into the cytosol and caspase activation, thus connecting the extrinsic to the intrinsic pathway of apoptosis [2]. The novelty of the current study resides in the demonstration that TRAIL and Bortezomib act together to cause the accumulation of tBid at the mitochondria [8]. To this end, tBid was found to accumulate at higher levels in cells that were treated with the combination of Bortezomib and TRAIL compared to cells that were exposed to TRAIL alone [8]. Since tBid has been reported to be prone to proteasomal degradation upon its ubiquitination [10], proteasome inhibition by Bortezomib prevents the degradation of tBid that is newly generated upon stimulation with TRAIL via TRAIL-induced caspase-8 activation, leading to the accumulation of tBid. This conclusion is supported by data showing that cells, which were treated with TRAIL to trigger the cleavage of Bid to its truncated form tBid, then washed to remove the remaining TRAIL and incubated in the presence of the broad-range caspase inhibitor zVAD.fmk to prevent any further processing of Bid by activated caspases, harboured markedly increased levels of tBid compared to cells, which were incubated in the absence of Bortezomib after the initial stimulation with TRAIL [8]. Since no tBid is produced any longer after removal of TRAIL and in the presence of the caspase inhibitor zVAD.fmk, neither by ongoing TRAIL stimulation (since TRAIL was removed by a washing step) nor by continued caspase activity (due to the addition of the wide-range caspase inhibitor zVAD.fmk), tBid levels under these experimental conditions are largely controlled by its degradation rate. Thus, the breakdown of tBid after TRAIL stimulation was substantially delayed in the presence of Bortezomib compared to cells that were incubated in the absence of Bortezomib after exposure to TRAIL. These findings support the conclusion that treatment with TRAIL results in cleavage of Bid into tBid, while the addition of Bortezomib markedly increases the stability of tBid that is produced by TRAIL-stimulated proteolytic cleavage. The functional relevance of tBid in the Bortezomib-conferred sensitization of neuroblastoma cells to TRAIL-induced apoptosis was demonstrated by RNA interference experiments showing that silencing of Bid also significantly reduced apoptosis in cells co-treated with TRAIL and Bortezomib [8]. Together, these results demonstrate that Bortezomib primes for TRAIL-mediated apoptosis at least in part by stabilizing tBid.

Various mechanisms of action have been proposed to mediate the cooperative action of TRAIL and Bortezomib in the induction of apoptosis in cancer cells, involving modulation of both the extrinsic and the intrinsic pathway of apoptosis. As far as the intrinsic apoptosis signaling pathway is concerned, Bortezomib has been reported to stimulate upregulation of the BH3-only domain proteins Bim and/or Bik [11, 12], to block the degradation of Bax [13] or to promote the release of Smac from the mitochondria into the cytosol [14]. Also, Bortezomib alone or in combination with TRAIL has been shown to downregulate or cleave anti-apoptotic Bcl-2 proteins such as Bcl-2, Bcl-X_L_ or Mcl-1 [15, 16]. It is interesting to note that Noxa turned out in the current study to determine only the kinetic of apoptosis induction, while it became dispensable for apoptosis upon prolonged exposure to Bortezomib and TRAIL [8]. In contrast, Noxa has been shown to be a critical mediator of apoptosis upon monotherapy with Bortezomib [17-19], pointing to differences in the role of Noxa between single agent and combination therapy with Bortezomib.

Bortezomib-mediated modulation of the extrinsic pathway of apoptosis has been attributed to upregulation of the agonistic TRAIL receptors TRAIL-R1 and/or TRAIL-R2 in several studies [16, 20-26], to promote the formation of the TRAIL DISC [27], to downregulate c-FLIP expression [27-29] or to block the degradation of caspase-8 [30]. Also, reduced expression of XIAP has been linked to Bortezomib-mediated sensitization to TRAIL-induced apoptosis [31-33] as well as inhibition of NF-κB by Bortezomib [24, 34].

In addition to providing new mechanistic insights into the synergistic interaction of Bortezomib and TRAIL the present study is the first demonstration that Bortezomib acts in concert with TRAIL in a childhood cancer, i.e. neuroblastoma. Previous studies on the combination of TRAIL and Bortezomib were performed in adult tumors. As single agent, Bortezomib has been reported to suppress neuroblastoma growth in preclinical studies [35-38], also in chemoresistant or metastatic disease [39-41]. By comparison, Bortezomib showed limited *in vitro* or *in vivo* activity as monotherapy when it was tested against the solid tumor cell line or xenograft panel of the pediatric preclinical testing program that includes models for neuroblastoma [42]. In phase I clinical trials in children with refractory solid tumors or leukemia Bortezomib proved to be well tolerated, although little single agent activity was reported [43, 44]. Together with our findings showing that Bortezomib sensitizes neuroblastoma cells towards TRAIL, the currently available data indicate that Bortezomib represents an interesting experimental agent for the treatment of neuroblastoma especially in combination protocols, which warrants further investigation.

**Figure 1:**
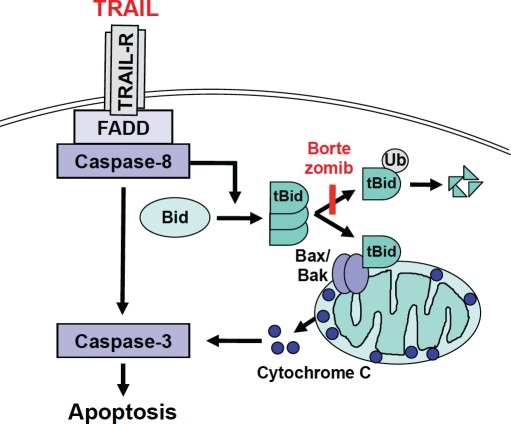
Scheme of the role of Bid for the synergistic interaction of TRAIL and Bortezomib TRAIL induces cleavage of Bid into tBid, while Bortezomib increases the stabilization of tBid by inhibiting its proteasomal degradation, resulting in accumulation of tBid at mitochondrial membranes. This in turn promotes activation of Bax/Bak, release of cytochrome c from mitochondria into the cytosol, caspase-3 activation and caspase-dependent apoptosis.
